# Translation and validation of the Arabic version of the Chronic Illness Anticipated Stigma Scale in Saudi patients with multiple sclerosis

**DOI:** 10.3389/fpsyt.2025.1443336

**Published:** 2025-02-21

**Authors:** Naseem A. Alhujaili, Salhah S. Alsulami

**Affiliations:** ^1^ Division of Psychiatry, Department of Medicine, Faculty of Medicine Rabigh, King Abdulaziz University, Jeddah, Saudi Arabia; ^2^ Department of Medicine, Faculty of Medicine Rabigh, King Abdulaziz University, Rabigh, Saudi Arabia

**Keywords:** chronic illness anticipated stigma scale, validation study, translation, noncommunicable diseases, Saudi Arabia, chronic diseases, Arabic language

## Abstract

**Background:**

Anticipated stigma is associated with experiences of enacted stigma or discrimination from others in the past or the present based on one’s chronic illness. People diagnosed with chronic diseases report experiencing significant stigma from others, including social rejection from friends and family members, work termination from employers, and poor care from healthcare providers. The aim of this paper was to explain the translation procedure and the psychometric evaluation of the Arabic language version of the Chronic Illness Anticipated Stigma Scale (CIASS), which was designed and evaluated psychometrically in different countries and languages.

**Methods:**

The Arabic version of the CIASS was given to 222 patients with multiple sclerosis (MS). Cronbach’s alpha was used to assess the internal consistency of the measured questionnaire. Structural equation modeling with confirmatory factor analysis using the maximum likelihood method was applied to evaluate the CIASS questionnaire in order to assess its factorial structure and validity for patients suffering from MS.

**Results:**

The Arabic version of CIASS is shown to have high reliability (Cronbach’s *α* = 0.89) and structural validity.

**Conclusion:**

The Arabic version of CIASS is a valid and reliable tool for the assessment of anticipated stigma among patients with MS. The use of this tool in the clinical setting can help to identify the source of stigma and guide its management accordingly. However, further validation studies among patients with different chronic illnesses are required.

## Introduction

Stigma is an “attribute that labels a person in discriminatory patterns.” Erving Goffman (1963) classically described stigma as a “deeply discrediting attribute.” Stigma is a sign that something about a person appears atypical (1). In 2001, Link and Phelan expanded the stigma model to include loss of social status and discrimination experiences (2). It has shifted from the kingdom of the well to the kingdom of the sick (3).

There are three types of stigmata: enacted, anticipated, and perceived. Anticipated stigma is the belief/perception of individuals that discrimination, prejudice, and stereotyping will occur to them in the future (4). Measures of anticipated stigma are significant if they consider specific sources of stigma. However, most stigma scales and tools only consider the extent to which stigma is anticipated from others in general, ignoring the origins of the stigma. It has been shown that stigma is experienced by specific groups, such as family members, employers, or healthcare workers (HCWs) (5).

Anticipated stigma is associated with experiences of enacted stigma or discrimination from others in the past or the present based on one’s chronic illness. People diagnosed with chronic diseases report experiencing significant stigma from others, including social rejection from friends and family members, work termination from employers, and poor care from healthcare providers (6).

People who have experienced stigma from others might expect to experience stigma in the future. In addition, anticipated stigma can also be created by the knowledge of the patient about negative stereotypes and attitudes in the community with regard to their illness, even without prior related negative experiences (6). People diagnosed with chronic diseases internalize stigma or devalue themselves due to their chronic condition. Internalized stigma is also related to anticipated stigma. This could be because how people see themselves often relates to how they think others see them (6, 7). Anticipated stigma negatively impacts the health of people living with chronic illnesses. People living with chronic diseases who anticipate stigma might socially separate themselves from friends and family members, preventing them from accessing critical social support that could benefit their health and wellbeing (8).

However, multiple pieces of evidence collected from various global settings have shown that chronic illness stigma negatively impacts people’s mental, behavioral, and physical health (9–11). In addition, stigma worsens chronic pain, disability, and social isolation (12). Furthermore, numerous researchers still seek to understand and address chronic illness stigma, which has been proven to improve the health of people living with chronic illness worldwide. The translation and validation of standard measures of stigma across global settings is an essential step toward this goal (9).

Hence, the development of the Chronic Illness Anticipated Stigma Scale (CIASS) was evaluated in the United States of America (USA) among people living with chronic conditions such as inflammatory bowel disease, multiple sclerosis (MS), fibromyalgia, and diabetes. The psychometric evaluation of the scale supports its reliability, validity, and generalizability in the USA. Moreover, CIASS scores have been associated with health behaviors, including accessing healthcare, and mental health, including depressive symptoms (4, 6, 13, 14). This paper aimed to explain the translation procedure and the psychometric evaluation of the Arabic language version of the CIASS as it was designed and evaluated psychometrically in different countries and languages; for example, the Persian and Spanish versions are recent ones (4, 15, 16).

## Methodology

### Study design and participants

The study was conducted in Saudi Arabia from July to September 2022. The Arabic version of CIASS was made into an online questionnaire, and links to the questionnaire were posted on social networking sites and messaging applications. All participants gave consent to participate in the study before filling out the questionnaire. The study excluded those not diagnosed with MS, those below the age of 18, those who are outside of Saudi Arabia, and those who refused to participate. A total of 222 patients diagnosed with MS were enrolled in the study and completed the online questionnaire. All of the patients reside in the Kingdom of Saudi Arabia and have undergone treatment for MS.

### Translation of the CIASS from English to Arabic

The CIASS was developed by Dr. V. Earnshaw and colleagues in 2013. It includes 12 items divided into three subtypes, the aim of which was to gauge the extent of anticipated stigma in patients with chronic illness from family and friends, work colleagues, and HCWs. The responses of the patients are on a Likert-type scale, ranging from very unlikely (1) to very likely (5).

The Arabic version of CIASS was translated from English to Arabic by a bilingual researcher and then back into English by two professional translators. Any discrepancies between the translations were reconciled. Both versions were evaluated by psychiatry professionals, and assurance of the terminology used was done. A pilot study was conducted on 20 participants who took the questionnaire and assessed its clarity afterward, and they were eliminated from the analysis.

### Data analysis

Cronbach’s alpha was used to assess the internal consistency of the measured questionnaire. All quantitative descriptive analyses were performed using IBM^®^ SPSS software, version 25. Internal consistency was assessed using Cronbach’s alpha, and values equal to or greater than 0.70 were considered to be satisfactory.

Structural equation modeling with confirmatory factor analysis (CFA) using the maximum likelihood method was utilized for the MS patients’ perceptions of the CIASS questionnaire in order to assess its factorial structure and validity. The parallel analysis (PA) test was used to assess the number of factors that may exist within the questionnaire. The closeness-to-unidimensionality of the 12-item-long CIASS questionnaire was determined with the Unico test to assess whether it can be treated as unidimensional.

### Ethical considerations

This study was approved by the research Ethics Committee at King AbdulAziz University, Jeddah, Saudi Arabia, with reference number 283-22.

The translation and validation study received permission from Dr. Valarie Earnshaw (developer of the CIASS tool). All participants were assured of confidentiality.

## Results

**Table 1** displays the descriptive analysis of the patients diagnosed with MS. The vast majority of patients (65.8%) were women, with the remainder (34.2%) being men. The mean ± SD age of the patients was 33.10 ± 8.58 years, with an age range of 17–60 years between the youngest and the eldest. Of the patients, 47.3% were single, 43.2% were married, and 9.2% were divorced. The education levels for the sample of patients were as follows: 2.7% had intermediate education, while 16.7% had completed secondary school. However, most of them (68.9%) had a university degree, while 11.7% had a higher study degree. The places of residence of the patients were distributed as follows: the majority of the patients (50%) are from western Saudi Arabia provinces, and 36.9% live in the central region of Saudi Arabia, where the capital city of Riyadh is based. Another 7.2% reside in the eastern part, while 5.9% are from the southern provinces.

**Table 1:** Descriptive analysis of the sociodemographic characteristics of the patients diagnosed with multiple sclerosis (*N* = 212).

	Frequency	Percentage
Sex		
Women	146	65.8
Men	76	34.2
Age group		
Age (years), mean (SD)		33.10 (8.58)
19–30 years	103	46.4
31–40 years	84	37.8
Over 40 years	35	15.8
Marital status		
Single	105	47.3
Married	96	43.2
Divorced	21	9.5
Education level		
Intermediate	6	2.7
Secondary	37	16.7
University	153	68.9
Higher studies	26	11.7
Residence		
Western provinces	111	50
Central region	82	36.9
Eastern provinces	16	7.2
Southern provinces	13	5.9
Have you been previously diagnosed with any mental illness?		
No	145	65.3
Yes	77	34.7
What mental illness do you have? (*n* = 77)		
Anxiety disorder	46	61.3
Depression	62	80
Delusional disorder	14	17.3
Obsessive–compulsive disorder	5	6.7
Other mental illness	4	5.3
How many episodes of MS illness did you have last year?		
One	114	51.4
Two	55	24.8
Three	34	15.3
Four	19	8.6
Can you do your activities of daily living (ADLs) without assistance from someone?		
I need assistance.	26	11.7
I can do ADLs without assistance.	196	88.3

The patients were asked to indicate (with yes/no) whether they had already been diagnosed with any mental/psychological illness. The majority of the patients (65.3%) advised that they had no prior diagnosis of mental illness, while 34.7% agreed that they had a prior diagnosed mental/psychological disorder. Approximately 61.3% of the participants with a prior mental/psychological disorder had anxiety disorders, 80% have been diagnosed with depression, 17.3% have been diagnosed with delusional disorders, and 6.7% with obsessive–compulsive disorders. A few of the patients (5.3%) have been diagnosed with other mental illnesses.

The patients were asked to indicate how many MS-related attacks they had experienced in the last 12 months. The analysis findings showed that more than half of the patients (51.4%) had experienced one MS episode, 24.8% had experienced two MS attacks, 15.3% had experienced three, and 8.6% had experienced four MS-related attacks. A total of 11.7% admitted needing assistance with their daily chores and activities of daily living (ADLs); however, most of them stated that they could do their ADLs independently.

### Descriptive analysis of the participants

**Table 2** displays the descriptive analysis of the MS patients’ perceptions of stigma as measured using the overall CIASS questionnaire. Each of the 12 indicators was measured with a likelihood rating scale graded as follows: 1 = very unlikely to 5 = very likely.

**Table 2:** Descriptive analysis of the multiple sclerosis patients’ perceptions of stigma.

	Mean	SD	Rank
Stigma from friends and family			
A friend or family member will be angry with you.	2.33	1.32	*1*
A friend or family member will blame you for not getting better.	2.32	1.31	*2*
A friend or family member will think that your illness is your fault.	1.76	1.1	*4*
A friend or family member will not think as highly of you.	2.24	1.25	*3*
Stigma from work colleagues			
Your employer will not promote you.	2.77	1.28	*3*
Someone at work will discriminate against you.	2.58	1.31	*4*
Your employer will assign a challenging project to someone else.	3.22	1.3	*2*
Someone at work will think that you cannot fulfill your work responsibilities.	3.29	1.29	*1*
Stigma from healthcare workers			
A healthcare worker will be frustrated with you.	2.15	1.16	*1*
A healthcare worker will give you poor care.	2.01	1.1	*3*
A healthcare worker will blame you for not getting better.	2.05	1.08	*2*
A healthcare worker will think that you are a bad patient.	1.89	1.06	*4*

#### Family and friends anticipated sigma

The MS patients’ top perceived anticipated family stigma indicator was anger of family members (mean score = 2.33/5). The second perceived family stigma source was that of one family member blaming them for not getting better (mean score = 2.32/5). The third source is that family members do not think as highly of the MS patients and pin the fault of their illness on the patients themselves (mean score = 1.67/5).

#### Colleagues and co-workers anticipated stigma

The top perceived anticipated coworker stigma indicator for MS patients was that people at the workplace think that the patient could not fulfill his/her work responsibilities and tasks (mean score = 3.29/5). This was followed by that of an employer/supervisor assigning challenging projects/assignments to another coworker instead of the patient himself/herself (mean score = 3.22/5), as well as not getting promoted by employers (mean score = 2.77/5). The least perceived anticipated stigma indicator was discrimination against the patients based on their illness.

#### Anticipated stigma from healthcare workers

The MS patients’ top expected HCW stigmatization was that of HCWs being frustrated with the patients themselves (mean score = 2.15/5) and then blaming them for not improving (mean score = 2.05/5), receiving poorer care than expected as a result (mean score = 2.01/5). The least perceived stigmatization by HCWs, according to the MS patients, was that of HCWs thinking that the patients are bad persons/patients.

### Measurement of reliability and validity

The Cronbach’s alpha for internal consistency was used to assess the reliability of the 12-item CIASS questionnaire when used in the Arabic-translated version for MS-diagnosed patients. The results of the analysis showed that the overall internal consistency of the questionnaire (12 items) was substantial, with a Cronbach’s alpha of 0.89. Furthermore, the four items comprising the patients’ perceived family stigmatization had great internal consistency (Cronbach’s *α* = 0.800). Similarly, the subscale of colleague/coworker stigmatization was measured as reliable (Cronbach’s *α* = 0.841). Moreover, the HCW anticipated stigma subscale items were measured to have substantive internal consistency. Overall, this indicates that the 12 items of the CIASS were read and understood by patients equally reliably (**Table 3**).

**Table 3:** Internal consistency and reliability analyses of the Chronic Illness Anticipated Stigma Scale (CIASS) .

	No. of items	Cronbach’s alpha
Stigma from friends and family subscale score	4	0.8
Stigma from work colleagues subscale score	4	0.841
Stigma from healthcare workers subscale score	4	0.89
Overall CIASS questionnaire	12	0.89

**Figure 1:**
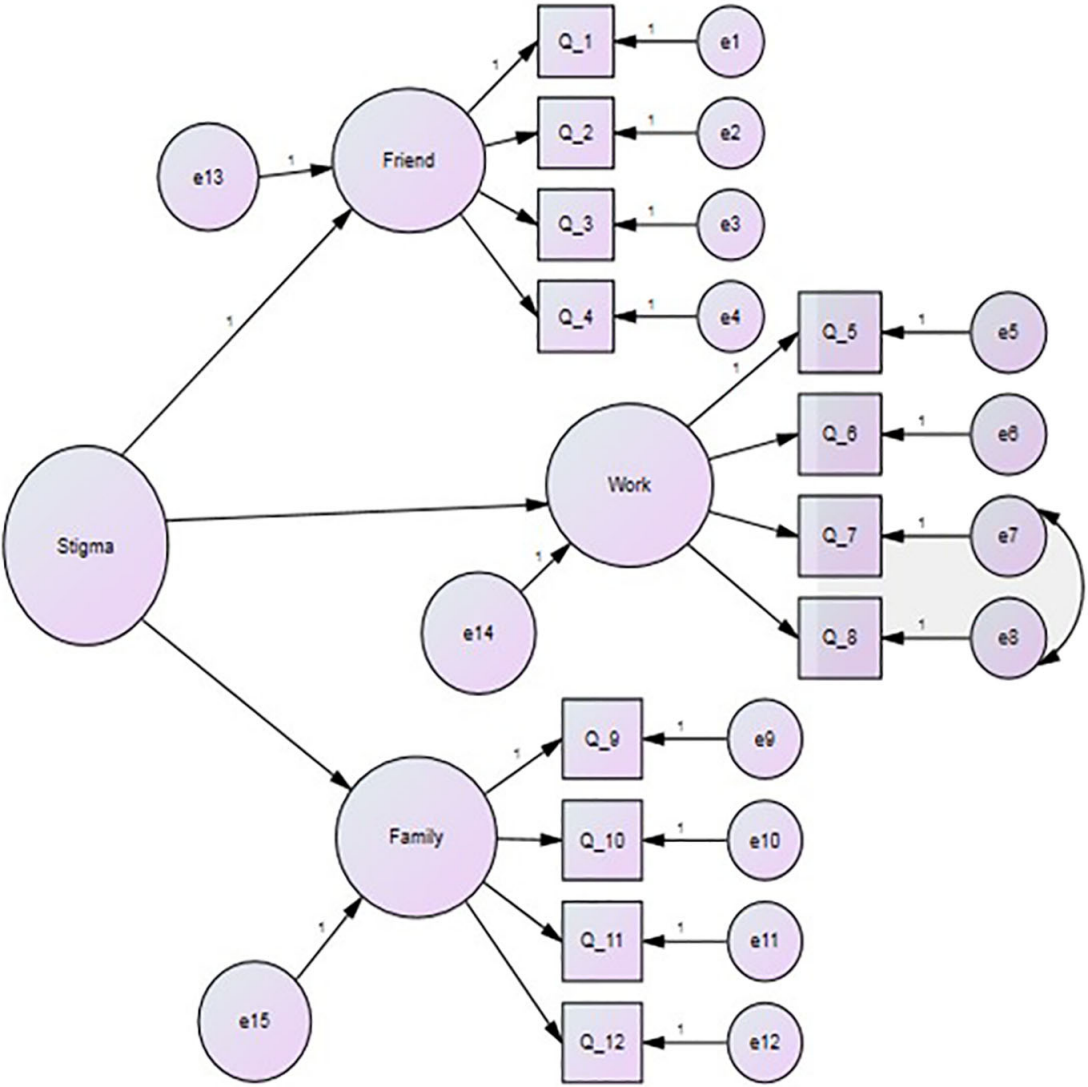
Confirmatory factor analysis (CFA) of the questionnaire.

The CFA of the second-order model for the 12-item CIASS questionnaire, as shown in **Figure 1**, greatly fitted the data. However, as initially examined, there were no covariances between the indicators. The statistical analysis program yielded modification indices that suggested a correlation between items 7 and 8, which measure employer discrimination and stigmatization, and this was acceptable for us to consider. The analysis was repeated, which yielded goodness-of-fit indices that showed a better fit of the proposed second-order model with the data: root mean square error approximation (RMSEA) = *C*_MIN_/*DF χ* ^2^ = 82.91, *p* = 0.002 (RMSEA = 0.055, 90%CI = 0.033–0.075, *p*_CLOSE_ = 0.339), comparative fit index (CFI) = 0.976, Tucker–Lewis index (TLI) = 0.968. All of these indices showed agreement on the goodness of fit of the factor analysis, except for the chi-squared test; however, it is not uncommon for the chi-squared test to show departures of fit in a big sample size, such as the one used in this study.

**Table 4** shows the standardized regression coefficients for the loadings of each item and the latent factors to their parent construct. Each of the subscales (friend-, coworker-, and family-related stigma) had loaded (i.e., correlated) saliently and significantly positively (>0.60 each) to the overall anticipated chronic illness stigma upper factor (*p* < 0.001 each). Each of the indicators comprising each of the subscales loaded significantly and positively to their designated subscale factors, indicating the factorial validity of the Arabic-translated version of the 12-item CIASS questionnaire.

**Table 4:** Second-order confirmatory actor analysis of the standardized regression coefficients.

Item	Standardized regression coefficient	*p*-value
Stigma		
Friend	0.833	<0.001
Work	0.895	<0.001
Family	0.633	<0.001
Friend-related stigma		
Item 1	0.718	<0.001
Item 2	0.754	<0.001
Item 3	0.639	<0.001
Item 4	0.742	<0.001
Coworker-related stigma		
Item 5	0.769	<0.001
Item 6	0.777	<0.001
Item 7	0.655	<0.001
Item 8	0.686	<0.001
Family-related stigma		
Item 9	0.838	<0.001
Item 10	0.815	<0.001
Item 11	0.823	<0.001
Item 11	0.84	<0.001

Model goodness-of-fit statistics: *C*_MIN_/*DF χ* ^2^ = 82.91; *p* = 0.002 (RMSEA = 0.055; 90%CI, 0.033–0.075; *p*_CLOSE_, 0.339); CFI, 0.976; TLI, 0.968.

Furthermore, the PA test (conducted using the stand-alone factor program) suggested the presence of one overall fact that can be extracted from the 12 items. This was congruent with the closeness-to-unidimensionality test (Unico = 0.96), which advised that the overall CIASS questionnaire can be essentially unidimensional. As such, the CFA findings were accepted. The three subscale scores (friend-, family-, and coworker-related stigma sources) explained 70.4% of the variations between the MS patients on their perceived disease-related stigma, which is a substantive amount of explained variance. The composite reliability for the questionnaire was substantial (*C*_r_ = 0.833). A mean score was computed by averaging the 12 questionnaire items, yielding a mean chronic illness stigma anticipated score between 1 and 5 points. This mean score was explored further using the standard multivariable linear regression analysis.

## Discussion

Professor Valerie Earnshaw and her team were the first to introduce the measurement of anticipated stigma associated with chronic illness among patients with a variety of chronic diseases (4). This original English CIASS version is a brief measure with good psychometric properties, as confirmed by validation studies in the USA, Iran, Ethiopia, Colombia, and Italy (4, 5, 15, 16).

There is growing interest worldwide in exploring anticipated stigma related to different chronic disabling diseases, including MS and major depressive disorder (17, 18).

This study aimed to translate, culturally adapt, and investigate the psychometric properties of the Arabic version of CIASS in Saudi patients with MS.

The findings support the overall and subscale reliability (i.e., internal consistency) of the Arabic version of the CIASS. The overall internal consistency of the questionnaire (12 items) was significant, with Cronbach’s alpha of 0.89, which is close to that of the original English version, i.e., 0.93. It was also higher than the Cronbach’s alpha of 0.81, which was obtained in a validation study conducted in Spanish among Colombian patients (16). Setting a standard translation method and executing the psychometric test daily were the two reasons for the observed consistency.

Structural validity was assessed using CFA, and it was discovered that, except for the chi-squared test, all of the indices showed agreement on the goodness of fit of the factor analysis. This was similar to the English version, where the chi-square value was small, but statistically significant (*χ* ^2^ = 88.59, *p* = 0.0008) (3). There was a positive correlation between the subscales related to family, friends, and coworkers and the overall chronic illness stigma scale.

In conclusion, anticipated stigma is a prevalent condition that patients with MS face in daily life. Moderate to severe anticipated stigma has been found in more than 70% of patients diagnosed with MS (17), the study of which is essential as it affects the health, behavior, and the response to treatment of people living with chronic illnesses. It is critical to tackle stigma in patients with lifelong illness as it could worsen their chronic pain, social isolation, and depression (12). Moreover, stigma was associated with a decrease in the quality of life and worsened the mental symptoms of patients with MS (18, 19). Recognizing the root causes of stigma is essential for policymakers to address in order to support chronically ill patients in becoming more functional and socially acceptable.

This is the first study to have translated and presented data on the validity of the CIASS in Arabic. The Arabic version of CIASS is a valid and reliable tool for the assessment of anticipated stigma among patients with MS.

The main strength of the study is its inclusion of a sample from various regions of Saudi Arabia. In addition, it is useful when time is of the essence. This tool is valid for utilization in a clinical setting and as a self-administered survey. Another strength of the study is that it provides a detailed anticipation of stigma that is classified into different domains to aid in identifying the source of stigma and to guide management.

However, this study was limited to patients with MS. As a result, the findings may not be applicable to other patients suffering from various chronic illnesses. Furthermore, the gender distribution among the study sample was not balanced; therefore, its generalizability should be addressed with caution.

Additional validation studies are required for other diseases, as well as the inclusion of a sample with larger number of participants with better gender balance.

## Data Availability

The raw data supporting the conclusions of this article will be made available by the authors, without undue reservation.
