# Supplementary material for: Translation and validation of the Arabic version of the Chronic Illness Anticipated Stigma Scale in Saudi patients with multiple sclerosis
Source: Front Psychiatry. 2025 Feb 21;16:1443336. doi: 10.3389/fpsyt.2025.1443336 (PMC11886633; doi:10.3389/fpsyt.2025.1443336)
Supplement: Supplementary file 1 [file Presentation1.pdf]

TRANSLATION AND VALIDATION OF THE ARABIC VERSION OF CHRONIC ILLNESS ANTICIPATED STIGMA SCALE (CIASS) IN SAUDI PATIENTS WITH MULTIPLE SCLEROSIS

BACKGROUND

Enacted stigma is common among chronic diseases. stigma can be from friends and family members, workplace, termination from employers, and poor care from healthcare providers.

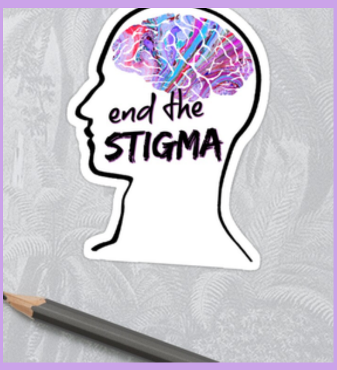

AIMS:

Translation procedure and psychometric evaluation of the Arabic language version of the CIASS

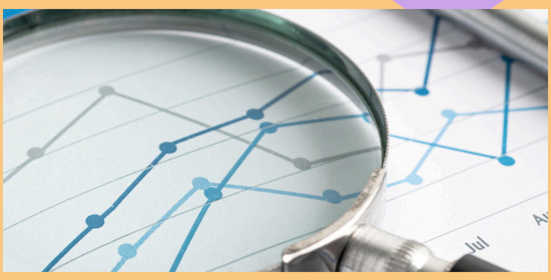

METHODS:

The Cronbach's alpha test used to assess the internal consistency. The structural equation modelling with confirmatory factor analysis using likelihood method used to assess CIASS

RESULTS:

Arabic CIASS among 222 patients with multiple sclerosis is a reliable, and structurally valid.

CRONBACH ALPHA: 0.89

CONCLUSION:

The Arabic CIASS is a valid and reliable tool for assessing anticipation stigma among MS patients. Using this tool in clinical settings can help to identify the source of stigma and guide the management accordingly.
